# Supplementary material for: Exploring the Links Among Risky Substance Use, Problematic Internet Use, and Academic Outcomes in University Freshmen: The Role of Mediating Factors
Source: Eur J Investig Health Psychol Educ. 2025 Jun 7;15(6):105. doi: 10.3390/ejihpe15060105 (PMC12191502; doi:10.3390/ejihpe15060105)
Supplement: Supplementary file 1 [file ejihpe-15-00105-s001.zip › ejihpe-3583915-supplementary.pdf]

Supplementary Materials

Table S1. Correlation matrix (Spearman rho's coefficients) among the first outcome (academic performance), substance/internet risky use, and academic engagement variables

		GPA	UCS	AMS_ Amotiv	AMS_ExtR	AMS_IntroR	AMS_IdentR	AMS_IntriR	SASP	ASSIST_ alcohol	ASSIST_ cannabis	ASSIST_ sedatives	IAUQ
GPA	Spearman's Rho	—											
	P	—											
UCS	Spearman's Rho	0.138	—										
	P	< .001	—										
AMS_Amotiv	Spearman's Rho	−0.198	−0.380	—									
	P	< .001	< .001	—									
AMS_ExtR	Spearman's Rho	−0.151	−0.378	0.726	—								
	P	< .001	< .001	< .001	—								
AMS_IntroR	Spearman's Rho	−0.111	−0.133	0.345	0.302	—							
	P	0.004	< .001	< .001	< .001	—							
AMS_IdentR	Spearman's Rho	0.159	0.329	−0.437	−0.346	0.205	—						
	P	< .001	< .001	< .001	< .001	< .001	—						
AMS_IntriR	Spearman's Rho	0.193	0.455	−0.450	−0.397	0.136	0.711	—					
	P	< .001	< .001	< .001	< .001	< .001	< .001	—					
SASP	Spearman's Rho	0.330	0.387	−0.331	−0.260	−0.102	0.340	0.439	—				
	P	< .001	< .001	< .001	< .001	0.006	< .001	< .001	—				
ASSIST_ alcohol	Spearman's Rho	−0.024	−0.096	0.055	0.061	0.066	−0.032	−0.068	−0.102	—			
	P	0.527	0.010	0.138	0.103	0.078	0.388	0.067	0.006	—			
ASSIST_ cannabis	Spearman's Rho	−0.064	−0.138	0.065	0.098	0.040	−0.095	−0.049	−0.105	0.284	—		
	P	0.098	< .001	0.082	0.008	0.279	0.011	0.187	0.005	< .001	—		
ASSIST_ sedatives	Spearman's Rho	−0.066	−0.094	0.150	0.112	0.105	−0.093	−0.070	−0.072	0.115	0.115	—	
	P	0.087	0.011	< .001	0.003	0.005	0.013	0.061	0.054	0.002	0.002	—	
IAUQ	Spearman's Rho	−0.100	−0.268	0.364	0.301	0.184	−0.143	−0.211	−0.303	0.128	0.098	0.083	—
	P	0.009	< .001	< .001	< .001	< .001	< .001	< .001	< .001	< .001	0.009	0.026	—

The higher and significant Rho values related to GPA outcome are highlighted in gray. Light gray: 0.1< absolute (Rho)<0.2; dark gray: absolute (Rho)>=0.2

Table S2. Correlation matrix (Spearman rho's coefficients) among the second outcome (dropout intention), substance/internet risky use, and academic engagement variables

		Dropout Intention	UCS	AMS_Amotiv	AMS_ExtR	AMS_IntroR	AMS_IdentR	AMS_IntriR	SASP	ASSIST_ alcohol	ASSIST_ cannabis	ASSIST_ sedatives	IAUQ
Dropout_ Intention	Spearman's Rho	—											
	p	—											
UCS	Spearman's Rho	−0.467	—										
	p	< .001	—										
AMS_Amotiv	Spearman's Rho	0.423	−0.380	—									
	p	< .001	< .001	—									
AMS_ExtR	Spearman's Rho	0.383	−0.378	0.726	—								
	p	< .001	< .001	< .001	—								
AMS_IntroR	Spearman's Rho	0.189	−0.133	0.345	0.302	—							
	p	< .001	< .001	< .001	< .001	—							
AMS_IdentR	Spearman's Rho	−0.351	0.329	−0.437	−0.346	0.205	—						
	p	< .001	< .001	< .001	< .001	< .001	—						
AMS_IntriR	Spearman's Rho	−0.453	0.455	−0.450	−0.397	0.136	0.711	—					
	p	< .001	< .001	< .001	< .001	< .001	< .001	—					
SASP	Spearman's Rho	−0.426	0.387	−0.331	−0.260	−0.102	0.340	0.439	—				
	p	< .001	< .001	< .001	< .001	0.006	< .001	< .001	—				
ASSIST_ alcohol	Spearman's Rho	0.164	−0.096	0.055	0.061	0.066	−0.032	−0.068	−0.102	—			
	p	< .001	0.010	0.138	0.103	0.078	0.388	0.067	0.006	—			
ASSIST_ cannabis	Spearman's Rho	0.100	−0.138	0.065	0.098	0.040	−0.095	−0.049	−0.105	0.284	—		
	p	0.007	< .001	0.082	0.008	0.279	0.011	0.187	0.005	< .001	—		
ASSIST_ sedatives	Spearman's Rho	0.131	−0.094	0.150	0.112	0.105	−0.093	−0.070	−0.072	0.115	0.115	—	
	p	< .001	0.011	< .001	0.003	0.005	0.013	0.061	0.054	0.002	0.002	—	
IAUQ	Spearman's Rho	0.228	−0.268	0.364	0.301	0.184	−0.143	−0.211	−0.303	0.128	0.098	0.083	—
	p	< .001	< .001	< .001	< .001	< .001	< .001	< .001	< .001	< .001	0.009	0.026	—

The higher and significant Rho values related to Dropout Intentions outcome are highlighted in gray. Light gray: 0.1< absolute (Rho)<0.2; dark gray: absolute (Rho)>=0.2

Figure S1. Baron and Kenny mediation model definition

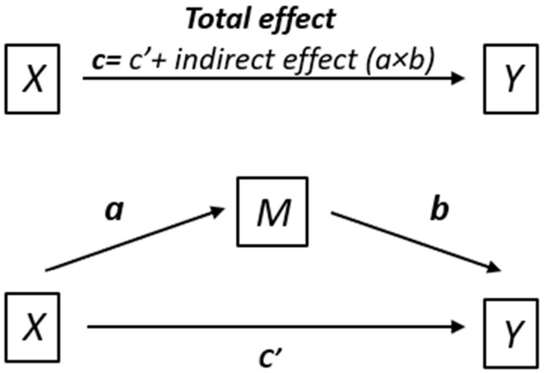

Mediation Model steps	
Step 1	Assessment of the association between X (independent variable) and Y (dependent variable): [c'] (direct effect)
Step 2	Assessment of the association between X and M (mediator): [a]
Step 3	Assessment of the association between M and Y (controlling for X on Y) [b]
Type of mediation	When the effect of M on Y is controlled:<br>i) Full mediation: if X is no longer associated with Y<br>ii) Partial mediation: if the association between X and Y is reduced
